# A Case of Severe Septicemia With *Chromobacterium violaceum* in Bangladesh

**DOI:** 10.1002/ccr3.70603

**Published:** 2025-07-11

**Authors:** Tuhin Sadique, Asif Reza Khan, Dilruba Ahmed, Fazlul Kabir, Anup Chowdhury, Nazrul Islam, Farhana Halim, Nasrin Akter, Shoeb Bin Islam

**Affiliations:** ^1^ Clinical Microbiology and Immunology Laboratory Office of Executive Director (OED), icddr,b Dhaka Bangladesh

**Keywords:** global health, health informatics, healthcare management, infectious disease, public health

## Abstract

*Chromobacterium violaceum* is a rare but potentially fatal pathogen found in tropical and subtropical regions. Infections with *C. violaceum* have rarely been reported in humans, with an exceptionally high mortality rate, particularly when linked to septicemia. We report the case of a 45‐year‐old female farmer from Bangladesh who developed severe septicemia following an insect bite and subsequent self‐drainage of an abscess. Blood cultures identified *C. violaceum* . The patient was treated empirically with intravenous ciprofloxacin and ceftriaxone, leading to full recovery despite resistance to ceftriaxone. Successful management of *C. violaceum* infections requires early diagnosis and prompt antimicrobial therapy. The intrinsic resistance mechanism of this organism, including the blaCVI gene associated with Carbapenem resistance, necessitates careful antibiotic selection. The literature review highlights successful cases, depending largely on timely intervention. This case highlights the need for careful antibiotic selection based on clinical response and sensitivity patterns. Moreover, increasing awareness among healthcare professionals, particularly in tropical and subtropical regions, is vital for early detection and successful treatment of this rare, life‐threatening infection.

**Summary**

- A 45‐year‐old woman from Bangladesh developed severe septicemia caused by *Chromobacterium violaceum* . Infections with this pathogen are rare but can be fatal and severe. Appropriate diagnosis, timely administration of the patient, and proper antibiotic selection are recommended to minimize the risks.

## 1 Introduction

*Chromobacterium violaceum* is a Gram‐negative and rod‐shaped bacterium that usually produces the distinctive pigment violacein [1]. It commonly resides in the soil and water of tropical and subtropical regions [2]. *Chromobacterium violaceum* was discovered in 1881, while in 1905, P.G. Woolley discovered its pathogen potential when he isolated it from a buffalo that had died from a lethal infection in the Philippines [3]. The first characterized case of *C. violaceum* infection in a human was on record in 1927 in Malaysia by J.E. Lessler [1]. Since then, over 160 human cases have been published in countries like India, Sri Lanka, Southeast Asia, Hong Kong, Australia, Brazil, and the United States [4]. Infections with *C. violaceum* have rarely been reported in humans with an exceptionally high mortality rate, particularly when linked to septicemia [5]. The combination of severe sepsis and inefficient antibiotic treatment resulted in fatality rates reaching up to 65% in reported cases [6]. We have reported a case of septicemia, which was caused by *Chromobacterium violaceum* . This is most likely the first successfully treated case of septicemia linked to *Chromobacterium violaceum* through blood culture in Bangladesh.

## 2 Case History/Examination

In July 2023, a 45‐year‐old female farmer from Narayanganj, Dhaka, Bangladesh, was admitted to icddr,b, Dhaka, Bangladesh. She presented with high‐grade fever, diarrhea, headache, chills, dizziness, jaundice, anorexia, and respiratory distress. Her medical history was unremarkable, with no known comorbidities such as diabetes or immunodeficiencies. Before admission, she had been in good health the previous month. While working in a paddy field, an unknown insect bit the patient on the left thigh. After the bite, the patient noticed abscess formation with swelling, erythema, and pain within 4 days. She tried to do self‐treatment at home. She used a stick of coconut leaf to incise the abscess and drained some pus from it. She added that she collected the stick from her yard. The abscess healed after 2 weeks. However, 21 days later, a high‐grade fever developed.

On admission, she appeared very sick with a temperature of 101.84°F, pulse rate of 145 beats/min, respiratory rate of 65 breaths/min, blood pressure of 80/60 mmHg, and bilateral crepitations. Laboratory studies showed a leukocyte count of 18,930/mm^3^ and a normal platelet count with 91% neutrophils, 03% lymphocytes, 02% eosinophils, and 04% monocytes. The ESR (erythrocyte sedimentation rate) was 93 mm/h. The blood hemoglobin was 10.9 g/dL. Creatinine was 150.442 μmol/L. Chest X‐ray revealed mild cardiomegaly and right‐sided basal pneumonitis. Moreover, random blood glucose levels were within the normal range. Additionally, she received oxygen inhalation for breathlessness. The ECG displayed T‐wave inversion in leads V1–V4. We suspected severe sepsis, and she was immediately started on intravenous ciprofloxacin 200 mg (every 12 h) and ceftriaxone 2 g (every 24 h) for a 7‐day course. Following the observation of heart failure (pro‐BNP 19265 pg/mL), she was immediately transferred to the National Institute of Cardiovascular Diseases (NICVD) the next day.

## 3 Differential Diagnosis

**FIGURE 1:**
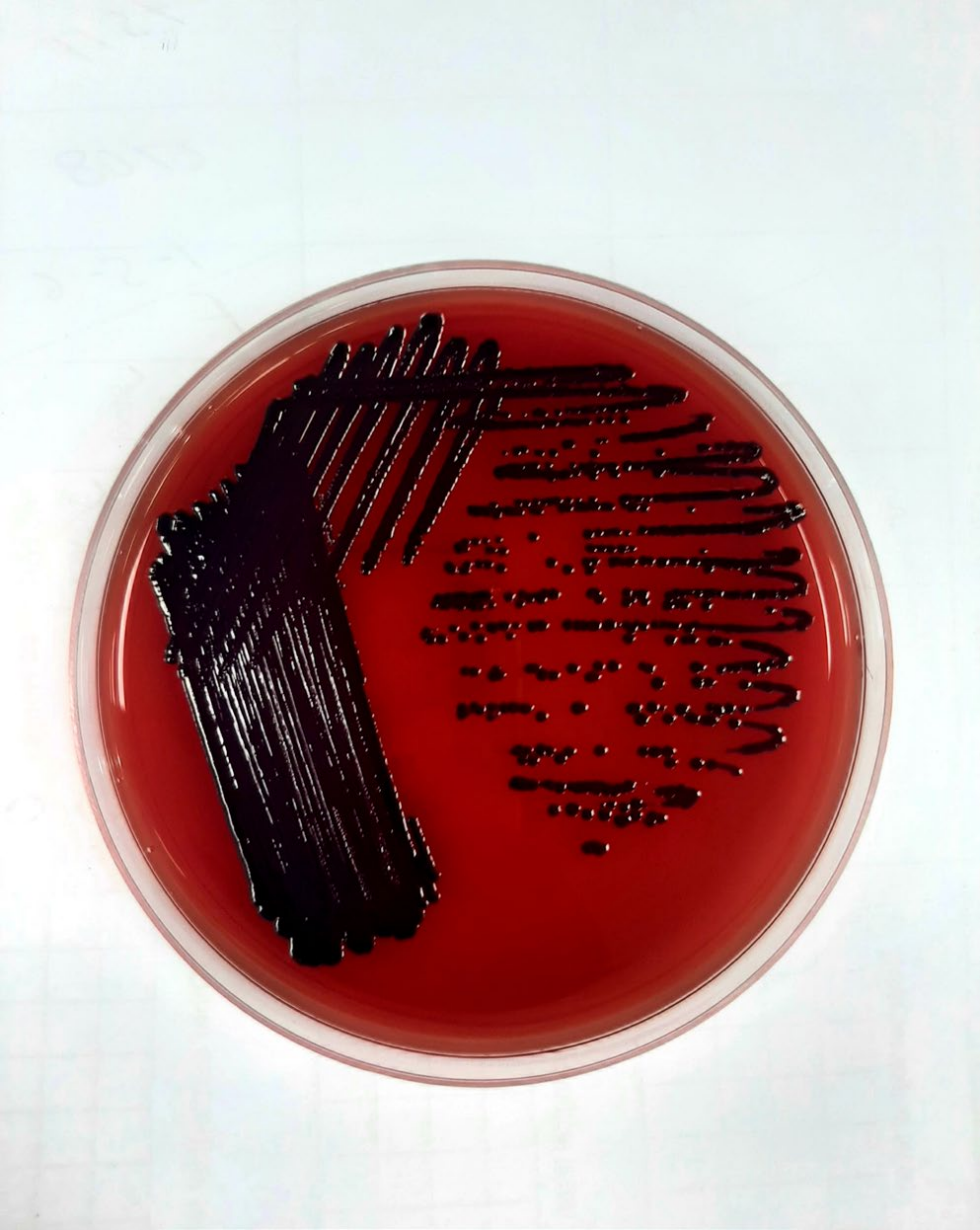
*Chromobacterium violaceum* produces dark violet‐pigmented colonies on a blood agar plate (referenced in Section 3).

Blood was collected into an aerobic blood culture bottle, and the culture was performed on the BacT ALERT 3D, which signaled positive. The blood was subcultured on MacConkey agar, Chocolate agar, and Blood agar (5% sheep blood). After overnight incubation, circular and smooth colonies with dark violet pigmentation were detected on MacConkey agar, Chocolate agar, and Blood agar plates (Figure 1). The Gram staining of the culture revealed the presence of Gram‐negative coccobacilli. The pathogen was identified as *C. violaceum*. The identification was confirmed with 98% probability using the Vitek2 system with the latest software update (Version 8.01) (Vitek2 Bio number: 5067000140541210). An antibiotic susceptibility test was performed using the disk diffusion method, using 13 different antibiotics, and the results were interpreted according to the CLSI guidelines for non‐lactose fermenting Gram‐negative bacteria. The isolate was sensitive to amikacin, ciprofloxacin, cefepime, gentamicin, and tigecycline, but resistant to amoxicillin–clavulanic acid, ceftriaxone, ceftazidime, cefuroxime, cefixime, colistin, meropenem, and piperacillin–tazobactam.

## 4 Conclusion and Results (Outcome and Follow‐Up)

After receiving the culture identification and antimicrobial susceptibility results, our clinician could not change the antibiotic course due to the absence of our patient. Then, we called the patient 3 months later, and she clarified that she had completed the previous 7‐day antibiotic course, which our clinician prescribed during her admission to the icddr,b hospital, and had responded better after the initial antimicrobial therapy. During a recent follow‐up, she was doing well, and her leukocyte, platelet, hemoglobin, and other blood cell parameters (Table 1) were within the normal range. An additional blood culture was also performed, and no bacterial growth was observed.

**TABLE 1:** List of laboratory findings (referenced in Section 4).

Tests	On admission	During follow‐up	Normal range
1. Leukocyte	18,930/cumm	6060/cumm	4000–11,000/cumm
2. Platelet	2,64,000/cumm	2,78,000/cumm	1,50,000–4,00,000/cumm
3. Neutrophils	91%	49.2%	40%–75%
4. Lymphocytes	03%	41.3%	20%–45%
5. Eosinophils	02%	5.9%	01%–06%
6. Monocytes	04%	3.3%	02%–08%
7. Basophils	00%	0.3%	00%–01%
8. Hemoglobin	10.9 g/dL	13.9 g/dL	12.0–16.5 g/dL
9. Creatinine	150.442 μmol/L	49.58 μmol/L	44–97 μmol/L

## 5 Discussion

Infections in humans caused by *C. violaceum* are rare [7]. In the recent decade, there has been a notable rise in the global occurrence of *C. violaceum* infections, especially in Southeast Asia, attributable to the recent shifts in global climate patterns [8]. *C. violaceum* is mainly found in tropical regions like South and Southeast Asia, and only three cases have been reported in Bangladesh before [9, 10, 11]. Still, there have not been any reported cases of confirmed septicemia linked to *C. violaceum* through blood culture in Bangladesh yet.

Healthy individuals usually get infected by this pathogen through drinking contaminated water, eating contaminated food, or exposing broken skin to stagnant water or contaminated soil. There have also been reported cases of severe infections following activities such as swimming in contaminated water, engaging in recreational activities in standing muddy water, and undergoing surgery [8, 12]. In our case, there is strong evidence indicating that the entry point was a skin injury, as she incised the wound with a stick of a coconut leaf. Perhaps the soil of the house‐yard could be a possible source of *C. violaceum* .

Infections caused by *C. violaceum* can lead to symptoms such as sepsis, liver abscess, kidney abscess, lung abscess, cellulitis at trauma sites, lymphadenitis, sinusitis, urinary tract infections, osteomyelitis, and meningitis. Sepsis is a common presentation, followed by lymphadenitis and liver abscess. Some cases may include pneumonia, diarrhea, or urinary tract infections. Skin manifestations are always linked with sepsis [13, 14, 15, 16, 17, 18, 19]. Our patient developed diarrhea, pneumonia, and acute respiratory distress syndrome with heart failure. Our patient had no pre‐existing diseases or predisposing factors. Although some patients with chronic granulomatous disease have experienced cases, suggesting it could be a contributing factor [20].

*C. violaceum* carries the blaCVI gene, which encodes a metallo‐β‐lactamase and contributes to its natural resistance to carbapenems, including meropenem [21]. This intrinsic feature should be considered when interpreting antimicrobial susceptibility results and guiding empirical treatment decisions. In a recent study by Gomez et al. (2023), a colistin‐resistant *C. violaceum* isolate from Argentina was found to harbor a novel β‐lactamase gene, blaCVI‐1, further highlighting the genetic versatility of this organism and its capacity to resist multiple antibiotic classes [21]. While our isolate did not undergo whole‐genome sequencing, the resistance profile—particularly to carbapenems and β‐lactam/β‐lactamase inhibitor combinations—supports the likelihood of similar underlying resistance mechanisms. These findings underscore the importance of continued surveillance and molecular characterization of *C. violaceum* better to understand its resistance determinants and potential clinical impact.

In our case, the patient showed clinical improvement with ciprofloxacin and ceftriaxone despite in vitro resistance to ceftriaxone. Similarly, other reports have documented successful outcomes using ciprofloxacin in combination with imipenem and piperacillin–tazobactam [22] or with meropenem [10]. Additional studies have demonstrated the effectiveness of ciprofloxacin either alone [23] or in combination with piperacillin [24] or trimethoprim–sulfamethoxazole [25]. These findings are consistent with the broad in vitro survey by Aldridge et al., which identified ciprofloxacin as the most effective agent against *C. violaceum* [26].

The general therapy for severe septicemia, particularly in ICU settings, requires prompt, empirical treatment due to the high mortality associated with delayed or inadequate therapy. Guidelines emphasize initiating broad‐spectrum antibiotics before culture results are available, as adjusting inadequate initial therapy later often does not improve outcomes. Empirical regimens should cover both Gram‐negative and Gram‐positive organisms [27].

This case is noteworthy because the patient had no immunocompromising conditions, and the infection was likely triggered by an unusual wound management method involving a coconut stick. Despite developing severe septicemia, the patient survived, aided by the timely initiation of appropriate antimicrobial therapy. Additionally, the absence of follow‐up during hospitalization highlights the importance of structured discharge planning and patient education.

This case highlights various essential aspects of septicemia induced by *Chromobacterium violaceum* . Infections with this pathogen are rare but can be fatal and severe. However, we suggest an appropriate diagnosis and timely administration of the patient, along with proper antibiotic selection, to reduce the severity of the infection. Enhancing awareness among healthcare professionals in tropical and subtropical regions is also essential.

## 6 Conclusion

This case highlights the need for careful antibiotic selection based on clinical response and sensitivity patterns. Moreover, increasing awareness among healthcare professionals, particularly in tropical and subtropical regions, is vital for early detection and successful treatment of this rare, life‐threatening infection.

## Author Contributions

**Shoeb Bin Islam:** conceptualization, data curation, formal analysis, writing – review and editing. **Asif Reza Khan:** data curation, investigation, writing – original draft, writing – review and editing. **Tuhin Sadique:** conceptualization, data curation, methodology, writing – review and editing. **Dilruba Ahmed:** conceptualization, investigation, resources, writing – review and editing. **Fazlul Kabir:** investigation, validation, writing – review and editing. **Anup Chowdhury:** formal analysis, methodology. **Nazrul Islam:** conceptualization, investigation, supervision, validation. **Farhana Halim:** formal analysis, software, writing – review and editing. **Nasrin Akter:** formal analysis, investigation.

## Consent

Written and informed consent was obtained from the patient to publish this case report. The written consent is available upon request if necessary.

## Conflicts of Interest

The authors declare no conflicts of interest.

## Data Availability

The data that support the findings of this study are available on request from the corresponding author. The data are not publicly available due to privacy or ethical restrictions.
